# iCogCA to Promote Cognitive Health Through Digital Group Interventions for Individuals Living With a Schizophrenia Spectrum Disorder: Protocol for a Nonrandomized Concurrent Controlled Trial

**DOI:** 10.2196/63269

**Published:** 2025-04-15

**Authors:** Christy Au-Yeung, Helen Thai, Michael Best, Christopher R Bowie, Synthia Guimond, Katie M Lavigne, Mahesh Menon, Steffen Moritz, Myra Piat, Geneviève Sauvé, Ana Elisa Sousa, Elisabeth Thibaudeau, Todd S Woodward, Martin Lepage, Delphine Raucher-Chéné

**Affiliations:** 1 Department of Psychology McGill University Montreal, QC Canada; 2 Department of Psychological Clinical Science University of Toronto Toronto, ON Canada; 3 Department of Psychology Queen's University Kingston, ON Canada; 4 Department of Psychiatry The Royal Institute of Mental Health Research University of Ottawa Ottawa, ON Canada; 5 Department of Psychoeducation and Psychology Université du Québec en Outaouais Gatineau, QC Canada; 6 Department of Psychiatry McGill University Montreal, QC Canada; 7 Douglas Mental Health University Institute Verdun, QC Canada; 8 Department of Psychiatry University of British Columbia Vancouver, BC Canada; 9 Vancouver Coastal Health Vancouver, BC Canada; 10 Department of Psychiatry and Psychotherapy University Medical Centre Hamburg Hamburg Germany; 11 Douglas Mental Health University Institute Montreal, QC Canada; 12 Department of Education and Pedagogy Université du Quebec à Montreal Montreal, QC Canada; 13 School of Psychology Université Laval Quebec, QC Canada; 14 BC Mental Health & Substance Use Services Vancouver, BC Canada

**Keywords:** schizophrenia spectrum disorders, digital technology, cognitive health, cognitive remediation, metacognitive training, schizophrenia, digital group interventions

## Abstract

**Background:**

Cognitive impairments are a key aspect of schizophrenia spectrum disorders (SSDs), significantly affecting clinical and functional outcomes. The COVID-19 pandemic has heightened concerns about mental health services and cognitive stimulation opportunities. Despite evidence-based interventions like action-based cognitive remediation (ABCR) and metacognitive training (MCT), a research-to-practice gap exists in their application across mental health settings.

**Objective:**

The iCogCA study aims to address this gap by implementing digital ABCR and MCT through a national Canadian collaborative effort using digital psychological interventions to enhance cognitive health in SSDs.

**Methods:**

The study involves 5 Canadian sites, with mental health care practitioners trained digitally through the E-Cog platform, which was developed by our research group. Over 2.5 years, participants with SSDs will undergo pre- and postintervention assessments for clinical symptoms, cognition, and functioning. Each site will run groups annually for both ABCR and MCT, totaling ~390 participants. A nonrandomized concurrent controlled design will assess effectiveness design, in which one intervention (eg, ABCR) acts as the active control for the other (eg, MCT) and vice versa, comparing cognitive and clinical outcomes between the interventions using generalized linear mixed effect modeling. Implementation strategy evaluation will consider the digital platform’s efficacy for mental health care practitioners’ training, contextual factors influencing implementation, and sustainability, using descriptive statistics for quantitative data and thematic analysis for qualitative data.

**Results:**

A pilot pragmatic trial has been conducted previously at the Montreal site, evaluating 3 early implementation outcomes: acceptability, feasibility, and engagement. Patient and therapist acceptability was deemed as high and feasible (21/28, 75% of recruited service users completed therapy, rated feasible by therapists). Technology did not appear to significantly impede program participation. Therapist-rated levels of engagement were also satisfactory. In the ongoing study, recruitment is underway (114 participants recruited as of winter 2024), and intervention groups have been conducted at all sites, with therapists receiving training via the E-Cog learning platform (32 enrolled as of winter 2024).

**Conclusions:**

At least 3 significant innovations will stem from this project. First, this national effort represents a catalyst for the use of digital technologies to increase the adoption of evidence-based interventions and will provide important results on the effectiveness of digitally delivered ABCR and MCT. Second, the results of the implementation component of this study will generate the expertise needed to inform the implementation of similar initiatives. Third, the proposed study will introduce and validate our platform to train and supervise mental health care practitioners to deliver these interventions, which will then be made accessible to the broader mental health community.

**Trial Registration:**

ClinicalTrials.gov NCT05661448; https://clinicaltrials.gov/study/NCT05661448

**International Registered Report Identifier (IRRID):**

DERR1-10.2196/63269

## Introduction

### Background

Schizophrenia and related psychoses present debilitating challenges, imposing an enormous burden on individuals, families, and communities [1,2] and are characterized by symptom recurrence, social deterioration, and cognitive impairments [3-8]. Most affected individuals experience persistent positive (eg, hallucinations and delusions) and negative symptoms (eg, amotivation, avolition, and reduced expressivity), alongside notable cognitive challenges like difficulties in verbal memory, executive functions, and attention. In addition to these impairments are cognitive distortions [9], affecting reasoning and information processing and, collectively, represent a core feature of schizophrenia, impacting clinical and functional recovery [10-13]. Thus, addressing overall cognitive health in schizophrenia is crucial. Over the past 25 years, advancements in psychological interventions, including cognitive remediation (CR) and metacognitive training (MCT), have shown promise for the treatment of schizophrenia spectrum disorders (SSDs). Both CR and MCT have been found to enhance cognitive functioning and decrease cognitive biases [14,15].

### Cognitive Health Interventions in Schizophrenia: State of the Evidence and Delivery Format

Meta-analyses affirm the effectiveness of CR in enhancing cognition [14,16,17] and MCT in addressing cognitive biases [15,18]. Notably, various mental health practitioners with an understanding of cognitive processes can be trained to provide these interventions, providing flexibility in service delivery [19-21]. Delivered in a group format, these interventions enable practitioners to reach multiple service users simultaneously [20]. Research indicates the feasibility of using technology for remote cognitive assessment and psychological interventions, as individuals with psychosis express interest and willingness to engage with digital mental health services [22-24] and find this mode of communication more satisfying and less challenging than observed in the general population [25].

### Preliminary Work on Remote Delivery of Cognitive Health Interventions

Our preliminary work investigated the remote delivery of action-based cognitive remediation (ABCR) and MCT [26]. ABCR is a distinct form of CR, which integrates the traditional cognitive training techniques of CR with simulated workplace scenarios and goal setting [27]. This work involved evaluating 3 crucial early implementation outcomes: acceptability, appropriateness, and feasibility for both patients and therapists. Across 6 cohorts (3 ABCR and 3 MCT) conducted within Montreal, patients expressed high acceptability, with overall satisfaction for expectations and perception of progress. The interventions demonstrated feasibility; 36 participants completed therapy, attending an average of 10 MCT sessions and 12 ABCR sessions.

Using recently developed measures [28], therapists also reported excellent acceptability, appropriateness, and feasibility of the interventions [26]. Several facilitators and barriers to delivering these digital interventions were also identified. Barriers comprised patients’ clinical status (eg, more severe symptomatology and medication side effects), interaction issues (eg, lack of involvement and decreased accountability), technological challenges (eg, access to devices and the internet), program elements (eg, language options), and scheduling conflicts. On the other hand, facilitators included patients’ motivation to learn, therapist characteristics (eg, warmth and proper training), financial support for internet connectivity, program support (eg, education on interventions and ice breakers), and logistical adjustments (eg, offering evening sessions and forming smaller groups).

### Objectives

Having established the acceptability, appropriateness, and feasibility of our digital interventions that collectively represent the early stages of implementation, the next step is to examine how the proposed digital strategies promote the uptake of these interventions across mental health care settings. A major challenge with evidence-based psychosocial interventions is that very few are subsequently tested in effectiveness or implementation trials and thus have little impact on population health [29]. The field of implementation science has emerged over the last 20 years, promoting strategies to adopt and integrate evidence-based interventions and change practice patterns within specific settings. Implementation frameworks, such as the Consolidated Framework for Implementation Research (CFIR) [30], provide the roadmap and tools to achieve this. To test this implementation strategy, we propose a study spanning 5 different mental health care sites across Canada. In collaboration with partners and knowledge users, we will conduct a hybrid effectiveness-implementation trial and create a bilingual digital learning platform (English and French) to ensure the long-term use of these digital interventions nationwide. The first objective of this study is to investigate the clinical effectiveness of the digital modality of ABCR and MCT. The second objective of the study is to investigate the implementation strategy involving (1) the contextual factors influencing the digital delivery of cognitive health interventions [31], (2) the effectiveness of a digital learning platform (E-Cog) to train mental health practitioners, and (3) the sustainability of the maintenance of these interventions within current clinical settings.

## Methods

### Study Design

A hybrid effectiveness-implementation trial [32-34] relying on digital technology will be used. The effectiveness component involves assessing the outcomes of these interventions, while the implementation component focuses on conducting the research in a way that emulates the naturalistic clinical setting. This design is ideal for transferring evidence-based behavioral interventions into real care environments [32-35], as it confirms clinical effectiveness while targeting necessary procedures to deliver and sustain such interventions in real-world care settings. A nonrandomized concurrent controlled design will be used to assess clinical effectiveness where one intervention acts as the active control for the other. The nonrandomized concurrent controlled design was selected, as it eases access to the preferred intervention by service users, overcoming the challenges of randomizing participants in real care settings and facilitating recruitment.

### Study Setting

This study takes place across 5 sites providing care to those with psychotic disorders across Canada. These sites include the Centre intégré universitaire de santé et de services sociaux (CIUSSS) de l’Ouest-de l’Ile de Montréal, Royal Ottawa Health Care Group, Kingston Health Sciences Centre, Ontario Shores Centre for Mental Health Sciences in Toronto, and Vancouver Coastal Health. We will recruit 390 service users across the 5 sites.

### Selection Criteria

Inclusion criteria include 18 years of age or older; diagnosis of affective or nonaffective psychosis or related disorder; followed and treated by a clinician at one of the services mentioned earlier; considered symptomatically stable and capable of using the digital platforms and participating in intervention groups, as judged by their primary clinicians; access to a private space to ensure group confidentiality; and provision of emergency contact and consent to allow researchers to contact their clinician or emergency services in the event of an emergency during study procedures. Most criteria are present for the safety of the group and participants. Exclusion criteria include intellectual disability, hospitalization at the time of recruitment, inability to speak or read English or French, and high suicide risk as per evaluation. We will recruit 4-6 mental health practitioners per site who will complete training on the E-Cog training platform to deliver the 2 cognitive health interventions. Practitioners will be eligible if they have a background in psychology, social work, nursing, or any other health-related training.

### Recruitment

Recruitment for this study uses a multimodal communication strategy designed to effectively reach potential participants. This strategy includes displaying informational flyers on hospital television screens, distributing email newsletters to health care employees, and presenting intervention details to case managers to facilitate referrals. Additionally, recruitment materials will be posted in clinic waiting rooms. n example of the recruitment pamphlet is provided in Multimedia Appendix 1. Health care team members can refer eligible participants or obtain consent for the research team to make direct contact. Participants are permitted to be engaged in other psychological or psychosocial interventions while participating in our study. Data on whether they are currently receiving other interventions will be collected.

### Ethical Considerations

This study has been approved by the respective research ethics boards (REBs) of the 5 mental health sites and their partner institutions. The study will be conducted in Montreal (CIUSSS de l’Ouest-de l’Ile de Montréal; REB 2023-561) [36], Ottawa (Royal Ottawa Health Care Group; REB 2022034) [37], Kingston (Kingston Health Sciences Center; REB 6037321) [38], Toronto (Ontario Shores Centre for Mental Health Sciences; REB 38116) [39], and Vancouver (Vancouver Coastal Health; REB H22-02300) [40]. The research adheres to the principles outlined in the Tri-Council Policy Statement: Ethical Conduct for Research Involving Humans and complies with Canadian legal requirements for scientific research involving human participants, including the Privacy Act and applicable clinical trial regulations [41]. Participants provide informed consent through a comprehensive process that includes a detailed explanation of the study’s objectives, procedures, potential risks and benefits, and their rights as research participants, in accordance with the Tri-Council Policy Statement: Ethical Conduct for Research Involving Humans guidelines. This process will involve both written documentation and verbal discussion, and participants will be informed of their right to withdraw from the study at any time without consequence to their ongoing care. Particular attention will be given to ensure an understanding of these processes among individuals with SSDs. All collected data will be deidentified using unique participant codes, with personal identifiers stored separately in encrypted files accessible only to authorized research personnel. In terms of compensation, participants will receive monetary remuneration for each completed assessment measure, with a standardized gift card amount of US $18 per assessment session or focus group. This compensation structure ensures fair and consistent reimbursement for participants’ time and effort while maintaining ethical standards for human participant research. The compensation details will be clearly communicated to participants during the informed consent process to ensure transparency and avoid undue influence on participation decisions.

### Measures

To assess the effectiveness of the 2 digital cognitive health interventions (objective 1), primary outcomes will encompass quantitative measures of cognitive function for ABCR and cognitive biases for MCT. These outcomes were selected in alignment with the primary targets for each intervention and primary outcomes assessed in the past ABCR and MCT studies. To capture the full extent of these outcomes, both objective and self-report measures were selected. The collection of these primary outcomes will be prioritized. Secondary outcomes for both interventions will include clinical and functional measures. Propensity score matching will consider background characteristics such as sociodemographic variables, illness duration, medication dosage, length and nature of prior treatment, and subjective reports of cognitive capacity and biases. To promote data quality, research staff will partake in centralized interrater reliability sessions every 6 months. To evaluate the implementation strategy (objective 2), a combination of quantitative and qualitative data will be gathered. Refer to Table 1 for a breakdown of the data types to be collected, organized by objectives, time points, and stakeholders.

**Table 1:** Summary of the type of data to be collected as a function of objectives, time points, and stakeholders.

Objectives or data type and measures			Measurement time points		Stakeholder groups
**1. Effectiveness (quantitative)**					
	Cognitive tests or tasks: Brief version of the CANTAB^a^ computerized battery (Intra-Extra Dimensional Set Shift, Stocking of Cambridge, and Spatial Working Memory)^b^ [42] Wechsler Memory Scale (Logical Memory Subscale)^b^ [43] Cognitive bias against disconfirmatory evidence story^b^ [44] Jumping to conclusions (Beads)^b^ [45] Subjective cognition: Subjective Scale to Investigate Cognition in Schizophrenia (Brief)^b^ [46] Cognitive Motivation Scaleb [47] Davos Assessment of Cognitive Biases Scale^b^ [48] Symptoms: Positive and Negative Syndrome Scale-6 [49] Brief Negative Symptom Scale [50] Psychotic Symptom Rating Scales [51] Psychosocial: Self-Esteem Rating Scale—Short Form [52] Overall Emotional and Social Loneliness Scale [53] Short Warwick-Edinburg Mental Well-Being Scale [54] Questionnaire About the Process of Recovery [55] Basic Psychological Need Satisfaction and Frustration Scale [56] Autonomous-Controlled Motivation for Intervention Questionnaire [57] Personal and Social Performance [58]	Preintervention Postintervention 3-month follow-up		Service users	
	Intervention: Health Care Climate Questionnaire [59] Autonomous-Controlled Motivation for Intervention Questionnaire [57] MMI^c^ Cognitive Training Questionnaire [60] Satisfaction With Therapy (STQ^d^) [61] Time spent on cognitive drill exercises (ABCR^e^) Last log in (iCog platform)	Midintervention		Service users	
**2. Implementation (quantitative and qualitative)**					
	Evaluation of E-Cog platform: Number of practitioners invited to participate in the E-Cog training Number of practitioners agreeing to participate in E-Cog training Reasons for nonparticipation or participation Number of practitioners not agreeing to participate in E-Cog training Total number of attendees participating in all of the training modules Total number of attendees per session or module Semistructured interview (health care practitioners)	Preintervention During intervention		Health care practitioners and service users	
	Evaluation of factors influencing the implementation of the 2 digital interventions: E-Therapy Attitudes and Process Questionnaire—Therapist Version [62] Semistructured interview (service users and health care practitioners)	Postintervention (8-12 and 24-30 months after the 2 interventions are implemented)		Health care practitioners and service users	
	Sustainability: Program Sustainability Assessment Tool [63] Monitoring of the intervention offer after an effectiveness trial at each site	Postintervention		Health care practitioners and service users	

^a^CANTAB: Cambridge Neuropsychological Test Automated Battery.

^b^Denotes primary outcomes in the study.

^c^MMI: MUSIC Model of Academic Motivation Inventory.

^d^STQ: Satisfaction Therapy Questionnaire.

^e^ABCR: action-based cognitive remediation.

### Cognitive Health Interventions

Interventions will be delivered through a secure videoconference platform. Network-connected tablets will be provided as necessary for the trial duration. ABCR [27,64] sessions consist of computer-based cognitive training activities (Brain Training Pro; 60%), teaching of problem-solving strategies (20%), and transfer activities (20%). Transfer activities include discussing and role-playing how cognitive skills are applied in everyday life and teaching potential strategies for overcoming cognitive challenges. ABCR targets include processing speed, attention, memory, executive functions, and social cognition, which are all commonly impaired in psychosis [12]. ABCR will be delivered in 16 sessions lasting 1.5 hours each over an 8-week period. MCT targets cognitive biases and errors in judgment underlying delusions using the theoretical foundations of cognitive behavioral therapy [65]. Sessions consist of discussions and activities aimed at increasing participants’ awareness of distortions and expanding their current problem-solving strategies. MCT will be delivered in 12 sessions lasting 45-60 minutes each over 6 weeks.

Both interventions will be offered at all sites. Depending on the site, interventions will either be offered concurrently or in sequence. Intervention group allocation follows a personalized, participant-centered approach. Potential participants will meet individually with the research coordinator at their site, who will provide a comprehensive overview of both interventions. Together, they will collaboratively determine which intervention best aligns with the participant’s personal goals and preferences. To maximize participant benefit and engagement, individuals will have the opportunity to complete the alternative intervention after finishing their initial chosen intervention. This flexible approach ensures that participants can potentially benefit from both interventions.

To improve adherence, participants will be contacted prior to each session with reminders. If participants miss several sessions in a row, therapists or a research assistant will contact them to confirm their interest in continuing the intervention and discuss any barriers to their participation. If any adverse reactions occur during the trial, participant involvement in our study may be discontinued after a discussion with the research team. Finally, to improve and track treatment adherence, sessions will be audio recorded, and 2 sessions from each of the ABCR and MCT groups will be randomly selected for review by 2 experienced facilitators using the treatment integrity assessment tool.

### Training Platform

E-Cog training platform [66] is a digital platform providing training certifications for ABCR and MCT interventions developed by our group following the analyze, design, develop, implement, and evaluate model for the design of digital learning platforms [67]. Each training certification includes three training modules: (1) impact of cognitive impairments in psychosis and an introduction to remediation strategies (2 hours), (2) technological tools for digital mental health (1 hour), and (3) theoretical foundations and practical delivery of ABCR (~9 hours) or MCT (~12 hours). After obtaining the training certification, mental health practitioners participate in weekly supervision by a dedicated experienced trainer using a secure videoconferencing software. Screenshots from the training platform can be found in the multimedia appendices (Multimedia Appendices 2-4).

### Involvement of Individuals With Lived Experience

Peer support workers have been involved at different stages of the project. The pilot study, which informed this study, involved 2 peer support workers in the project committee. In this study, our peer support worker has been providing continuous consultation, which has led to changes throughout the study (eg, adapting intervention materials). Our peer support worker will also be involved in the intervention groups and will be coconducting the service user qualitative interviews.

### Statistical Analysis

#### Power

Monte Carlo simulations computed in R (R Foundation for Statistical Computing) were used to estimate the required sample size for our proposed models. Our analyses, based on simulated data, suggest that a total sample size of 300 provides enough statistical power (up to 90%) to detect anticipated effect sizes on primary outcomes of cognitive capacity and cognitive bias based on values from our group and those reported in the literature (CR: *d*=0.50 and MCT: *g*=0.27) [9,20,27,31]. The attrition rate for our digital groups has been approximately 20%; we will nonetheless conservatively adjust for an attrition rate of 30%. When considering the propensity score, this results in a requirement of 390 participants. Comparable studies using the same interventions and statistical methods in in-person settings have included similar sample sizes as those proposed at our individual sites [68,69]. Further, this sample size will allow us to detect anticipated effect sizes on secondary outcomes related to symptomatology (CR: *d*=0.28 and MCT: *g*=0.38) [70,71] and functioning (CR: *d*=0.36-0.51 and MCT: *d*=0.37) [31,71,72]. We also anticipate that the proposed sample size will be adequate to explore sex- and gender-related differences using subgroup analyses of 2 (male and female) and 4 (men, women, nonbinary, and other) groups, respectively.

#### Data Analysis

Data related to the analysis of objective 1, focusing on clinical effectiveness, will be compared between both interventions. Primary and secondary outcomes will be compared between the 2 interventions, using one as the active control for the other. First, propensity score matching will be used to identify a subset of participants who will comprise the active control group that is equivalent to the intervention group on background variables (sociodemographic variables, illness duration, medication dosage, length and nature of prior treatment as well as subjective reports of cognitive capacity and cognitive biases). Then, Z-standardized outcome data will be compared between the groups with generalized linear mixed effect modeling techniques using R software. Factors of interest will include fixed time (pre, post, and follow-up), fixed treatment (ABCR and MCT), fixed time and treatment interaction (time*treatment), random site (CIUSSS de l’Ouest-de l’Ile de Montréal, the Royal’s Institute in Ottawa, Kingston Health Sciences Centre, Ontario Shores Centre for Mental Health Sciences in Toronto, and Vancouver Coastal Health), random intercepts (participants’ ID), and random slopes (participant*time). Age, illness duration, medication dosage, length, and nature of prior psychological treatment will also be included as fixed covariates. This procedure will be done twice, with the active control group subset using propensity score matching. This approach ensures that participants are not counted twice and is an integral part of the propensity score matching procedure. Statistically, performing the procedure twice helps to validate the robustness of the matching process without introducing bias, as each participant is only included once in each comparison. Specifically, the procedure will be as follows: (1) ABCR as the intervention and MCT as the active control and (2) MCT as the intervention and ABCR as the active control. Missing data pattern will be assessed for whether it is missing completely at random, missing at random, or not at random [73]. If data are missing at random, multiple imputation will be applied [74]; if data are missing not at random, then the robustness of the primary analyses will be evaluated through sensitivity analyses.

Analyses for all implementation objectives will be executed using descriptive statistics for quantitative data and thematic analysis for qualitative data, following Braun and Clarke’s [75] interpretive descriptive approach. This method emphasizes the researcher’s active role in the analytical process, allowing for a nuanced understanding of themes such as facilitators and barriers to implementation, identified from CFIR semistructured interviews. Investigator triangulation will be used, with 3 independent raters conducting iterative qualitative analyses until a consensus (≥80%) is reached. A convergent quantitative-qualitative mixed methods design [76] will facilitate a comprehensive comparison of results from quantitative and qualitative analyses, aiming to develop a holistic understanding of factors influencing implementation. Side-by-side comparison tables will support this analytical process and engage implementation teams in interpretation. To ensure reflexivity, member checks will be conducted with participants to validate findings, ensuring that their perspectives and interpretations are accurately represented and that any potential biases in the researcher’s analysis are addressed. This methodological framework is designed to uphold rigorous standards in qualitative research while ensuring that the findings are credible, dependable, and relevant to broader contexts. All qualitative analyses will be done using NVivo (version 15; Lumivero).

## Results

A pilot pragmatic trial [26] has been conducted previously at the Montreal site, where 6 cohorts (3 ABCR and 3 MCT) were run, evaluating 3 early implementation outcomes: acceptability, feasibility, and engagement. Of the 28 participants attending at least 1 session, 21 completed more than half of the sessions. All completers reported a positive experience with therapy, 2 of 3 were not bothered by the remote setting, and 16 trusted the confidentiality of the information shared. Technology did not appear to significantly impede program participation. Therapist-rated levels of engagement were also satisfactory [26].

This study was approved by the institutional review board by September 2023. From September 2022 to January 2023 staff training was conducted across all 5 sites. Intervention materials including intervention manuals and digital portals began in June 2022 and continued until November 2023. Therapist training began on the E-Cog learning platform in April 2023 and is ongoing. All sites began recruitment in 2023 and have run cohorts of MCT and ABCR in their respective regions. Quantitative data collection occurs prior to and following the intervention group and is ongoing, qualitative data collection for implementation assessment began in summer 2024 and is ongoing. As of winter 2024, a total of 114 participants have been recruited and 32 therapists are enrolled in the E-Cog training platform with 25 therapists having completed the training. The updated project timeline can be accessed for those who create free accounts on the iCogCA Hub web page [77].

## Discussion

### Overview

This study aims to address the crucial clinical needs of individuals with SSD by evaluating the feasibility and effectiveness of delivering cognitive health interventions through a digital platform. We hypothesize that these interventions will yield positive outcomes in terms of accessibility, engagement, symptom reduction, and cognitive functioning.

### Principal Findings and Comparison to Prior Work

The objectives of this study are 2-fold, focusing on both service users and mental health care providers. The first objective aims to assess the effectiveness of digital cognitive health interventions in enhancing accessibility and engagement for individuals with SSD. If proven effective, these interventions may provide an evidence-based framework for other service providers providing care for individuals with psychotic disorders and could be adapted for other populations receiving mental health services where these interventions show similar efficacy (eg, mood disorders) [78,79]. Objective 2 aims to identify facilitators and barriers to digital delivery and a digital training platform for clinicians. We anticipate that this study will introduce and validate our platform to train and supervise mental health care practitioners to deliver these interventions, which will then be made accessible to the broader mental health community. Further, these findings will furnish crucial information for the implementation of digital health technologies to improve access to psychosocial therapies for optimized care. These findings will be used to guide the implementation of similar projects across Canada. Notably, New York State has already fully integrated CR into its psychiatric care systems [80], and parallel efforts are underway in Australia [81] and the United Kingdom [82]. Collaborating with a National Steering and Implementation Committee, we will test our set of tools based on the CFIR and make them accessible to the mental health community. Site-specific implementation committees, which include patient partners, will assist with the local implementation of our cognitive health interventions.

### Strengths and Limitations

This proposed national collaborative effort serves as a catalyst for leveraging digital technology to enhance the adoption of evidence-based psychosocial interventions. This study has several strengths. The first is the implementation across multiple Canadian sites, which enhances the generalizability of our findings. Second, we also aim to engage a broad pool of participants, both service users and mental health practitioners, by using digital technology to provide the intervention and training. Encouragingly, previous studies have demonstrated that a significant portion of those with severe mental illnesses appear to have the required technological access and ability to join, participate, and benefit from digital services [83,84]. We have also a planned comprehensive collection of quantitative and qualitative data. Our data collection plan involves service users and mental health practitioners at multiple time points, allowing us to answer our research questions in a multifaceted manner. Finally, we have engaged with diverse stakeholders throughout various stages of the study, including those with lived experiences, clinicians, researchers, and managers.

Some limitations of our study include the nonrandomized concurrent controlled design, which raises concerns about potential group differences. However, we will address this by statistically controlling for measurable background characteristics. Second, although we aim to engage a broader population by providing services using a digital format, we acknowledge that there could be variability to technology access. We have planned to provide tablets and internet to service users without access to technology. For service users or health care providers unfamiliar with technology, we will also offer orientation sessions to help them become comfortable with the digital format. Next, the recruitment of therapists within a research setting may not reflect therapists involved in nonresearch settings. In addition, although we have taken great care in selecting instruments that have been validated in our population and available in both English and French, some limitations may still exist with the instruments chosen. For instance, ceiling effects have been observed in the Cambridge Neuropsychological Test Automated Battery cognitive test. Finally, there may exist differences across 5 sites in training experiences and implementation fidelity. We plan to attend to this by standardizing training and fidelity checks conducted across all sites.

### Conclusions

In summary, this project aims to address pressing clinical needs related to cognitive impairments and biases in SSDs. By leveraging digital technologies, it aims to facilitate the adoption of evidence-based interventions within clinical settings, support future implementation of similar initiatives, and validate an adaptable platform designed for widespread use.

## Appendix

Multimedia Appendix 1 Example of iCogCA recruitment pamphlet.

Multimedia Appendix 2 E-Cog training platform home page.

Multimedia Appendix 3 E-Cog training platform action-based cognitive remediation introduction page.

Multimedia Appendix 4 E-Cog training platform metacognitive training introduction page.

## Abbreviations

ABCR: action-based cognitive remediation
CFIR: Consolidated Framework for Implementation Research
CIUSSS: Centre intégré universitaire de santé et de services sociaux
CR: cognitive remediation
MCT: metacognitive training
REB: research ethics board
SSD: schizophrenia spectrum disorder

## Data Availability

The datasets generated or analyzed during this study are not publicly available due to ongoing data collection but are available from the corresponding author on reasonable request.
