# Supplementary figures and images for: iCogCA to Promote Cognitive Health Through Digital Group Interventions for Individuals Living With a Schizophrenia Spectrum Disorder: Protocol for a Nonrandomized Concurrent Controlled Trial
Source: JMIR Res Protoc. 2025 Apr 15;14:e63269. doi: 10.2196/63269 (PMC12041826; doi:10.2196/63269)

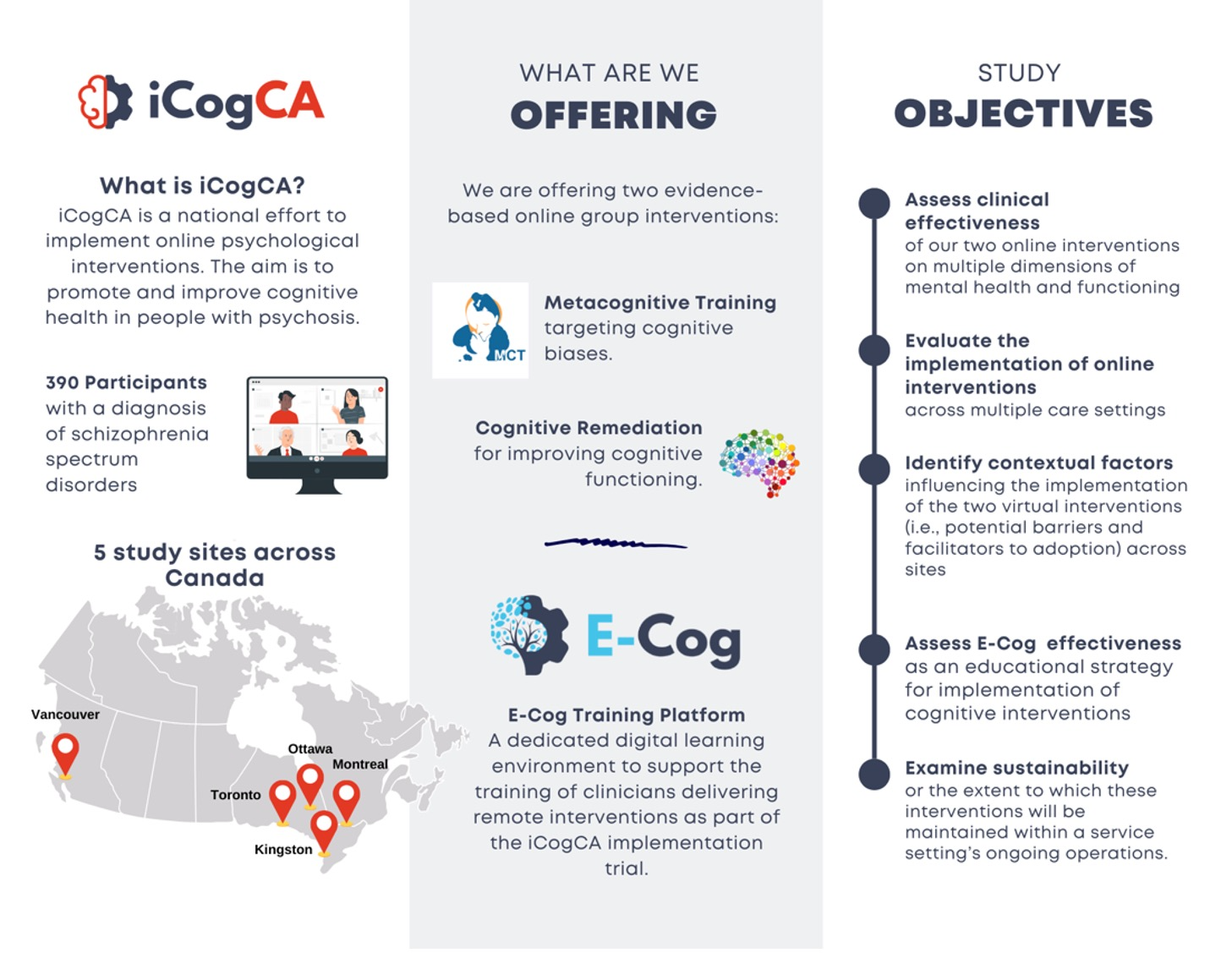

Supplement: Multimedia Appendix 1 [file resprot_v14i1e63269_app1.png]

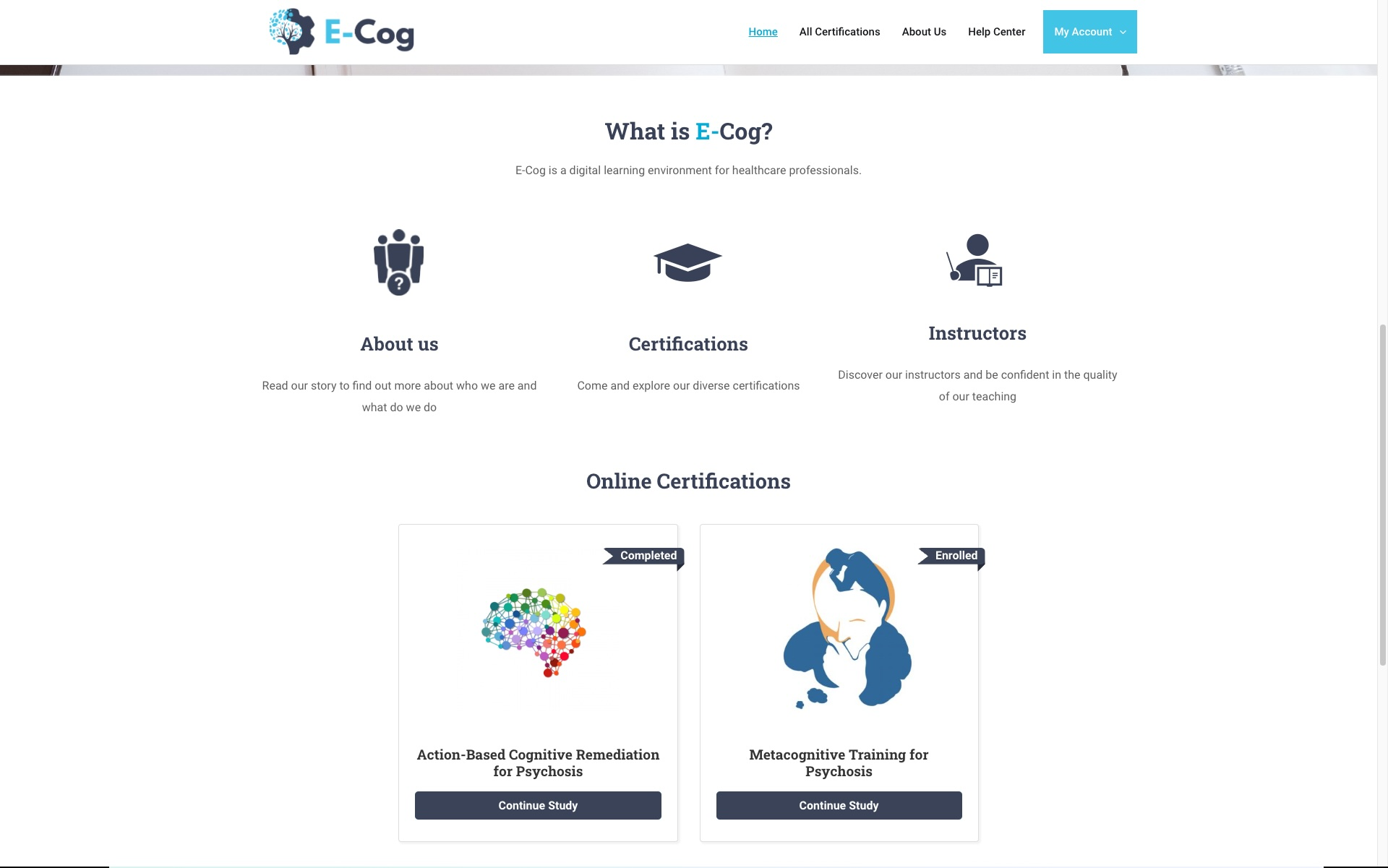

Supplement: Multimedia Appendix 2 [file resprot_v14i1e63269_app2.png]

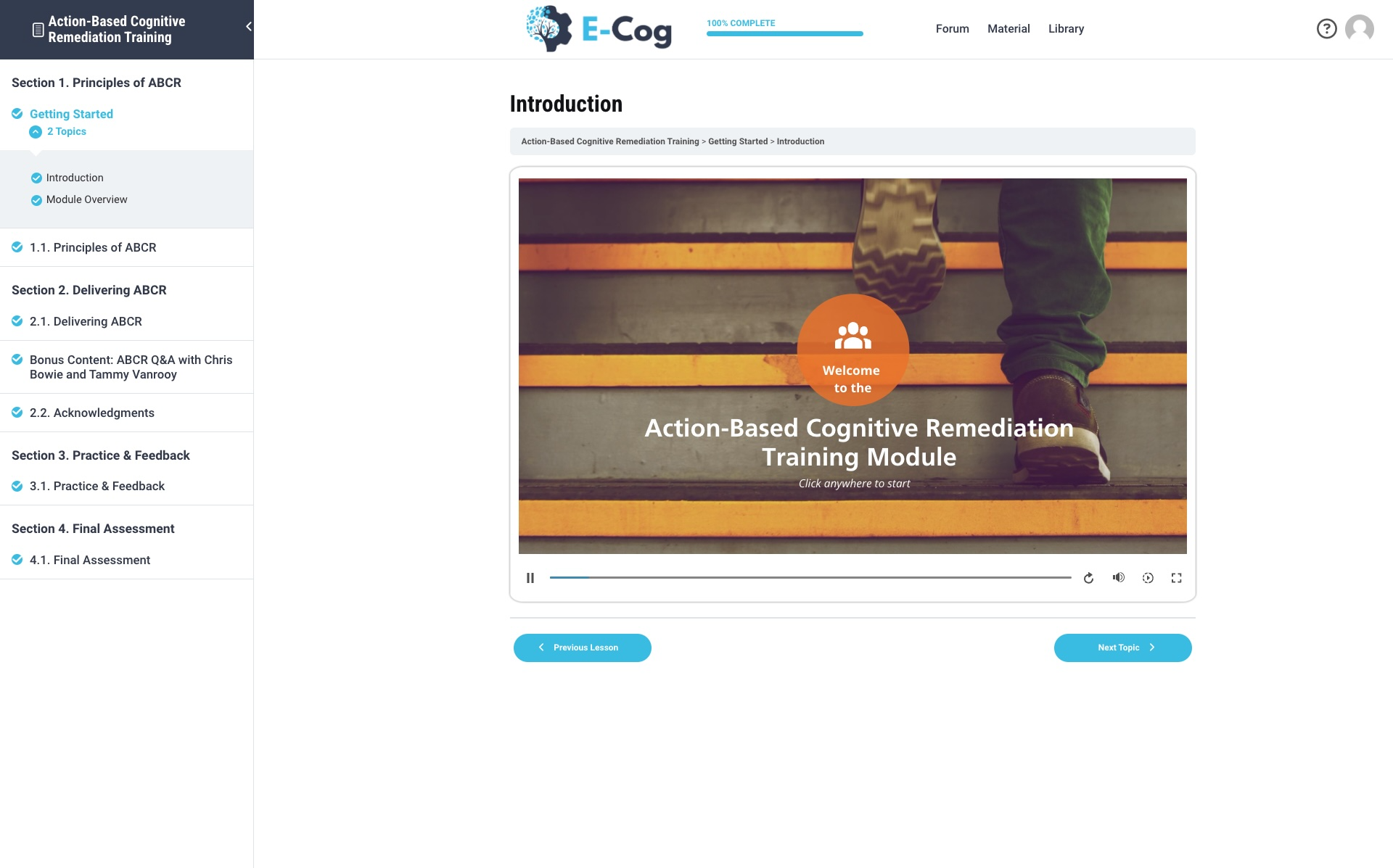

Supplement: Multimedia Appendix 3 [file resprot_v14i1e63269_app3.png]

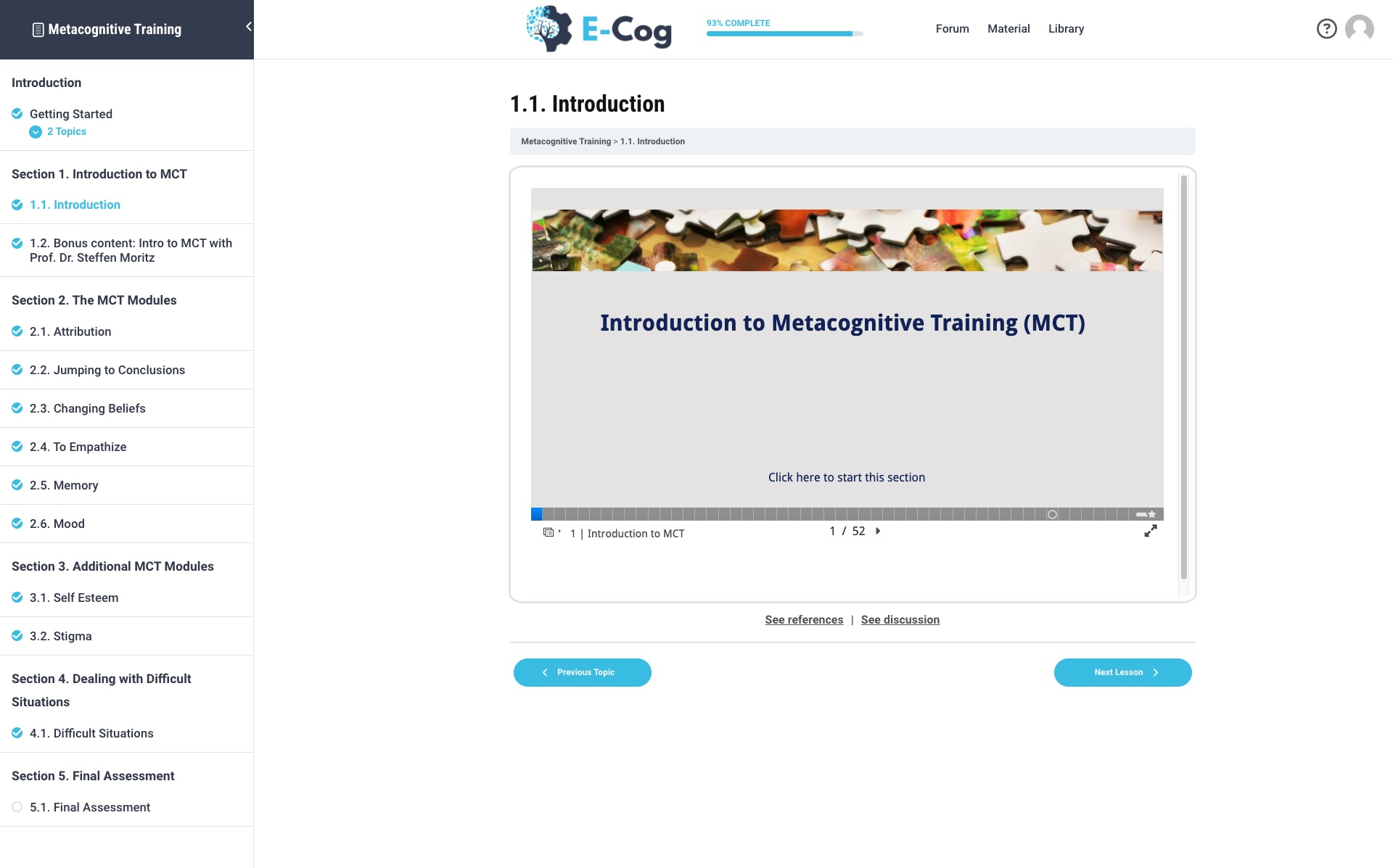

Supplement: Multimedia Appendix 4 [file resprot_v14i1e63269_app4.png]
